# Intrinsic and extrinsic factors affecting the evolution of virulence in the HIV-associated opportunistic human fungal pathogen *Cryptococcus neoformans*

**DOI:** 10.1080/21505594.2025.2546067

**Published:** 2025-08-14

**Authors:** Yuanyuan Wang, Yiwu Yu, Jihong Liu, Linghua Li, Xiaoqing Chen, Sichu Xiong, Yuying Pan, Qinyu Tang, Munika Moses, Ping Zhan, Guojian Liao, Min Chen, Liping Zhu, Muyuan Li, Tao Zhou, Jingjun Zhao, Changbin Chen

**Affiliations:** a Joint Laboratory for Biomedical Research and Pharmaceutical Innovation, Unit of Pathogenic Fungal Infection & Host Immunity, Key Laboratory of Molecular Virology and Immunology, Shanghai Institute of Immunity and Infection, Chinese Academy of Sciences, Shanghai, China; b Department of Dermatology, Tongji Hospital, Tongji University School of Medicine, Shanghai, China; c Department of Dermatology, Huadong Hospital, Fudan University, Shanghai, China; d Institute of Infectious Disease, Guangzhou No.8 People’s Hospital, Guangzhou, China; e University of Chinese Academy of Sciences, Beijing, China; f Seventh People’s Hospital, Shanghai University of Traditional Chinese Medicine, Shanghai, China; g College of Pharmaceutical Sciences, Southwest University, Chongqing, China; h Department of Dermatology, Shanghai Key Laboratory of Medical Mycology, Changzheng Hospital, Second Military Medical University, Shanghai, China; i Department of Infectious Diseases, Huashan Hospital, Fudan University, Shanghai, China; j Department of Dermatology, Xinhua Hospital, School of Medicine, Shanghai Jiaotong University, Shanghai, China; k Nanjing Advanced Academy of Life and Health, Nanjing, China

**Keywords:** *Cryptococcus neoformans*, virulence, genotype-phenotype correlation, environmental factors, genetic variants

## Abstract

The fungus *Cryptococcus neoformans* is considered the leading cause of mortality in immunocompromised patients. While extensive research has unveiled the molecular epidemiology of *C. neoformans*, the influence of genetic and environmental factors on genotype–phenotype correlations remains poorly understood. Specifically, it remains unclear whether the genetic and environmental variability observed across isolates from diverse sources has significant implications for the pathogen’s virulence. In this study, we analyzed 105 Chinese *C. neoformans* isolates, including 54 from HIV-infected patients, 44 from HIV-uninfected individuals and seven from a natural environment. Multilocus sequence typing (MLST) revealed that sequence type (ST) 5 predominates across all clinical isolates; however, genotypic diversity was notably higher in isolates from HIV-uninfected individuals and the natural environment, whereas HIV-infected isolates exhibited restricted genetic variation. Furthermore, isolates from HIV-uninfected individuals exhibited significantly enhanced virulence traits, including elevated capsule production, increased melanin production, improved survival in human cerebrospinal fluid (CSF), reduced phagocytic uptake, and higher mortality in both *Galleria mellonella* and murine models of cryptococcosis. Importantly, these pathogenic phenotypes were correlated with CD4^+^ T cell counts, highlighting the critical role of host immunity in shaping *C. neoformans* virulence. Whole-genome sequencing and genome-wide association studies (GWAS) further revealed that variations in genes involved in carbohydrate metabolism, such as *CDA3* and *GPD1*, may drive host-specific virulence evolution. Our results support a genotype–phenotype correlation, demonstrating that both genetic and environmental factors shape the virulence of *C. neoformans*, with significant implications for understanding host–pathogen interactions and guiding therapeutic strategies.

## Introduction

Cryptococcosis is a major invasive fungal infection caused by the encapsulated yeast species of the genus *Cryptococcus*, particularly *Cryptococcus neoformans* and *Cryptococcus gattii*. *C. neoformans* contains two varietal forms, *C. neoformans* var. *grubii* and *C. neoformans* var. *neoformans*, and is distributed worldwide. In contrast, *C. gattii* initially thought to be confined to tropical and subtropical regions, has now been recognized in temperate climates due to an outbreak on Vancouver Island, Canada [1]. The prevalence of cryptococcosis has been rising globally, with nearly 1 million cases of cryptococcal meningitis diagnosed annually, predominantly among immunocompromised patients due to Human Immunodeficiency Virus (HIV) infection, organ transplantation, chemotherapy, and corticosteroid use. Clinical isolates of *C. neoformans* and *C. gattii* species are responsible for 15% to 28% of deaths in HIV/AIDS patients due to cryptococcal meningitis. Meanwhile, *C. neoformans* accounts for the most common cause of meningitis in HIV adults in sub-Saharan Africa [2].

Phylogenetic and genotyping studies of clinical and environmental *C. neoformans* isolates worldwide have laid the foundation for understanding its global genetic structure [3], and significant genetic diversities are observed in the *C. neoformans* species complex. Initially treated as a single species with a conserved name [4], *C. neoformans* was later classified into four serotypes (A, B, C and D) based on Cryptococcal antigenic heterogeneity [5]. Revised taxonomy further categorized the serotype B and C isolates to *C. gattii* [6], while *C. neoformans* retained three major serotypes including *C. neoformans* var. *grubii* (serotype A), *C. neoformans* var. *neoformans* (serotype D) and the hybrid serotype AD [7]. Moreover, the *C. neoformans* species complex consists of two evolutionary divergent species, *C. neoformans* and *C. deneoformans*, as well as their associative hybrids (*C. neoformans* x *C. deneoformans*). Of the two lineages, *C. neoformans* exhibits a worldwide distribution and is responsible for approximately 95% of cryptococcal infections globally, with more than 99% of infections in AIDS patients, whereas *C. deneoformans* is more commonly found in Europe and is generally less virulent [8]. Due to the rapid development of molecular biology techniques, including PCR fingerprinting, amplified fragment length polymorphism (AFLP) analysis, MLST, and whole-genome sequencing [9], the ability to differentiate molecular types of the *Cryptococcus* genus at the molecular level has significantly improved. It is now well appreciated that *C. neoformans* can be classified into at least five major molecular types (AFLP1/VNI, AFLP1A/VNB/VNII, AFLP1B/VNII, AFLP3/VNIII and AFLP2/VNIV) [10]. Among these, *C. neoformans* var. *grubii* (serotype A/VNI) is commonly found in avian excreta and trees, exhibits a worldwide distribution, and contributes to over 80% of cryptococcosis cases [11]. Like the VNI clade, the VNII clade also has a global distribution, though *C. neoformans* var. *grubii* (serotype A/VNB) is predominantly found in sub-Saharan Africa and South America [12]. Moreover, *C. neoformans* var. *neoformans* (serotype D/VNIV) is mainly found in Western Europe and South America, while the hybrid *C. neoformans*(serotype AD/VNIII) is most prevalent in the Mediterranean region of Europe [13,14]. These findings collectively suggest that molecular types of the *C. neoformans* isolates not only differ in their serological, epidemiological and ecological characteristics, but also exhibit significant variation in clinical presentations, antifungal susceptibility and therapeutic outcomes.

Recently, a growing body of evidence have increasingly highlighted the impact of *C. neoformans* genotype on the host disease outcomes. For example, an epidemiological study by Wiesner *et al*. revealed that genotypes of *C. neoformans* isolates from HIV/AIDS patients in Uganda clustered into three distinct clonal groups within the VNI clade, with isolates ST93 and ST77 exhibiting the highest mortality risk [15]. Similarly, a study of clinical isolates from South African HIV/AIDS patients identified that ST32 isolates in the VNB clade correlated with poorer patient outcome [16]. Moreover, genotypic analysis of clinical *C. neoformans* isolates from Brazil revealed that ST93 in the VNI clade was the most prevalent sequence type in HIV-infected individuals [12]. However, a contrasting pattern was observed in Asian countries, where *C. neoformans* infections often occur in immunocompetent individuals [17], with ST5 being the predominant sequence type in *C. neoformans* isolates from East Asian countries including China, Japan, Vietnam and South Korea [18]. For example, a study using 136 Vietnamese clinical isolates of *C. neoformans* var. *grubii* revealed that ST5 isolates are responsible for 82% of infections in HIV-uninfected patients, compared to only 35% cases in HIV-infected patients [18]. In addition, MLST analysis of Chinese clinical *C. neoformans* isolates by different research groups also identified ST5 as the predominant sequence type [19]. Nevertheless, notable exceptions exist. For instance, studies have indicated that ST4 and ST6 are the major MLST types in *C. neoformans* var. *grubii* isolates of Thailand whereas ST93 turns to be the dominant isolates from India and Indonesia [20,21]. Interestingly, detailed information regarding genetic diversity of the isolates suggests that those from Thailand posit an evolutionary origin in African and the strains from China may have the same African origin but were expanded more flexibly and globally [21,22]. Taken together, these studies highlight the presence of global genetic diversity of *C. neoformans* isolates and their association with patient phenotypes. However, it is important to note that the pathobiological variations of *C. neoformans* isolates cannot be fully explained by genotypic diversity alone, and other factors should also be considered.

The factors determining disease prevalence and species specificity remain largely unknown, though they are speculated to be influenced by the host immune response, genetic variations, and virulence factors. For instance, the type 1 helper T-cell (Th_1_) response could stimulate classical macrophage activation and eliminate internalized cryptococcal cells. In contrast, the type 2 helper T-cell (Th_2_) response has been found to promote disseminated and uncontrolled cryptococcal infection [23,24], suggesting that patients’ immune responses to cryptococcal infection may lead to varied clinical outcomes. Furthermore, the distribution and prevalence of molecular types seem to be influenced by geographical factors, sample size and host characteristics [7,13]. For example, previous studies have shown that *C. neoformans* serotype A is one of the most common varieties and accounts for the majority of cryptococcal infections in Asia, especially in HIV-AIDS patients [23,24]. In addition, various virulence factors, including the presence and size of the polysaccharide capsule, melanin production by laccase, cell size variation, growth at 37°C and secretion of enzymes such as phospholipase, proteinase and urease, as well as sphingolipid utilization, have been linked to *C. neoformans* pathogenicity [25,26]. However, the correlation between these factors remains uncertain, and if such a correlation exists, the precise mechanisms by which they drive the evolution of *C. neoformans* pathogenicity are still not fully understood.

In an attempt to interpret the molecular epidemiology of *Cryptococcus* species, particularly the strain-level differences in genotype and phenotype, we investigated the genetic and environmental factors influencing the evolution of fungal virulence. A comprehensive analysis was conducted on a collection of 105 clinical and environmental isolates of *C. neoformans* from China, including both HIV-infected (HIV-i) and HIV-uninfected (HIV-u) patients. Genotypic characterization of each isolate was performed using MLST, while pathogenic phenotypes were rigorously assessed through *in vitro* and *in vivo* experiments. Furthermore, genotype–phenotype correlations were examined through genome-wide linkage and association studies. Our findings critically compare the relative impacts of genetic and environmental factors on the genotype–phenotype relationship, providing *in vitro* and *in vivo* evidence that underscores the significant role of both genetic and environmental influences in shaping the pathogenicity and genetic diversity of *C. neoformans* isolates.

## Materials and methods

### Ethics statement

All animal experiments were conducted in accordance with the Regulations for the Care and Use of Laboratory Animals established by the Ministry of Science and Technology of the People’s Republic of China, ensuring the ethical treatment of animals. The experimental protocol was approved by the Institutional Animal Care and Use Committee (IACUC) at the Shanghai Institute of Immunity and Infection, Chinese Academy of Sciences (Permit Number: 160651A). This study was conducted in compliance with the ARRIVE guidelines to ensure transparent and complete reporting of research involving animals.

This study was reviewed and approved by the Ethics Committee of Guangzhou No.8 People’s Hospital (20171491), Huashan Hospital, Changzheng Hospital and Southwest University (2018–010–01-KY). Studies involving human participants were conducted following the ethical principles outlined in the Declaration of Helsinki. Written informed consent was obtained from all subjects.

### Animals

Female C57BL/6 mice (6–8 weeks old, weighing 18–20 g) were purchased from Beijing Vital River Laboratory Animal Technology Company (Beijing, China). The mice were routinely maintained in a pathogen-free animal facility at a temperature of 21 °C, relative humidity of 50–70%, and under a constant 12-h light/dark cycle. Mice were given free access to food and water throughout the study. All procedures were conducted in compliance with a protocol approved by the IACUC at the Shanghai Institute of Immunity and Infection, Chinese Academy of Sciences, China.

### Strains

A total of 105 isolates of *C. neoformans* strains were included in this study, comprising 44 strains from the HIV-u patients, 54 from HIV-i patients, and 7 from the environment (Env group). Clinical and laboratory records for all patients were obtained from Guangzhou No.8 People’s Hospital, Huashan Hospital, Changzheng Hospital and Southwest University. The collected data for analysis included patient age, gender, initial symptoms, HIV infection status, and CD4^+^ T cell count (at the time of diagnosis). Among these isolates, 98 were obtained from cerebrospinal fluid (CSF) samples (*n* = 90) and blood cultures (*n* = 8). The *C. neoformans* clinical isolates were purified as single colonies on YPD medium and stored as glycerol stocks at −70°C for long-term preservation. Prior to experimentation, each strain was streaked and grown as a single colony on yeast peptone dextrose (YPD) medium prior to use.

A list of reference strains was included in this study, consisting of international strains used for phylogenetic analysis (H99 from USA, WM148 and WM626 from Australia, ST93 from Brazil), as well as standard strains for *in vitro* and *in vivo* assays (JEC20 and H99). All strains were cultured on yeast peptone dextrose (YPD) medium prior to use.

To construct the *GPD1*^OE^ overexpression strain, we use a plasmid containing homologous arms of Safe Heaven region in *Cryptococcus neoformans*, the PCnH3 overexpression promoter as previously described [27]. We amplified the GPD1 CDS region from the H99 genome and cloned the fragment into the vector via homologous recombination (Vazyme, C112–01). The plasmid was delivered into the H99 strain via electroporation and spread the product into G418 Resistant plate. To confirm the successful integration at the SH2 locus, we performed PCR verification using primers CTGTAGAAGAGCGAATAACCTT and AGCTGTGACAACTTGGC which target regions flanking the SH2 homologous arms. Additionally, we designed primers ATGTCTTTCCGAGTCCCTAC and CCTGGGCATCGAAGATAGA for qRT-PCR to assess the expression level of *GPD1* relative to H99. The strain with the highest *GPD1* expression level was selected for subsequent experiments. All the primers used in this study are listed in Table S2.

### DNA extraction

Isolates were cultured on Sabouraud dextrose agar slants for 48 hours. Single colonies were picked, re-inoculated in 10 ml of liquid medium, and incubated at 30°C for 24 hours. The cells were collected by centrifugation, and genomic DNA was extracted using the cetyltrimethylammonium bromide (CTAB) DNA isolation method as previously described [28].

### RNA extraction and RT-qPCR analyses

Total RNA was extracted following the procedure described previously [29]. Subsequently, 1 μg of total RNA was reverse transcribed using the PrimerScirpt RT Kit (Takara). qPCR was performed using the SYBR Premix Ex Taq II (Takara). The primer sequences used for qPCR are listed in Table S2. Glyceraldehyde-3-phosphate dehydrogenase (GAPDH) gene was used as an internal control for normalization of expression levels. All experiments were performed in triplicate.

### Multilocus sequence typing (MLST)

Multilocus sequence typing (MLST) was performed using the seven ISHAM consensus loci (*CAP59*, *GPD1*, *IGS1*, *LAC1*, *PLB1*, *SOD1*, *URA5*), following a standard procedure [30,31]. All the primers used in this study have been previously described [15] and are listed in Table S2.

### Assays for melanization and capsule formation

Melanin production was assessed by comparing the pigmentation of strains grown on L-DOPA medium to reference strains with strong (H99) and weak (JEC20) melanization, using the method described previously [32]. The relative melanization scores were assigned as follows: zero (equivalent to JEC20), one to four (between JEC20 and H99), and five (equal to or greater than H99), based on comparison with the reference strains grown on the same plate.

The capsule induction assay was performed as previously described [33], with slight modifications. Briefly, stationary-phase fungal cultures were washed and resuspended in PBS, diluted 1:100 in capsule induction medium [10% Sabouraud dextrose medium in 50 mM MOPS buffer (pH 7.3)]. The cultures were incubated at 30 °C with shaking at 180rpm for 48 hours. Capsule size was measured by staining the cells with India ink and imaging them at a magnification of X63 under a light microscope. The diameters of the entire cell (yeast cell + capsule) and the cell body (the cell wall only) were measured using Image J software, with additional measurements were performed using Adobe Photoshop software (Adobe Inc, USA). The data was further analyzed by a K-means clustering algorithm to classify capsule size variations. The mean values for 10–20 cells per isolate were calculated.

Mating type identification was performed via classical PCR analysis using mating type- and serotype-specific primers listed in Table S2.

### Infection of macrophages with cryptococcus

Phagocytosis assays were conducted using the murine macrophage-like cell line J774. Briefly, macrophage cells were cultured in DMEM supplemented with 10% heat-inactivated fetal bovine serum (FBS), 1 mM L-glutamine, and 1% penicillin–streptomycin (Sigma-Aldrich) in a 24-well tissue culture plate fat 37° C with 5% CO_2_ for 24 hours. After this, 1.5 × 10^5^ macrophages were incubated in serum-free DMEM for 2 hours, followed by activation with 15 μg/ml phorbol myristate acetate (PMA) (Sigma-Aldrich) for 30–60 minutes. Subsequently, the cells were co-incubated with *C. neoformans* yeast cells opsonized by a monoclonal antibody (C66441M, purchased from Meridian Life Science, Inc) for 2 hours at 37°C with 5% CO_2_ (MOI = 1:10). After incubation, extracellular yeast cells were removed by extensive washing with pre-warmed PBS. The extent of phagocytosis was determined by counting the number of cryptococci internalized by macrophages 2 hours post-infection. Data are presented as the mean of 3 to 4 independent experimental replicates.

### CSF survival assay

Human cerebrospinal fluid (CSF) was obtained from patients undergoing serial therapeutic lumbar punctures at Shanghai Changzheng Hospital. Pooled samples from at least 15 patients were collected anonymously. The human CSF was thoroughly examined to ensure parameters, including white cell count, protein, and glucose levels, were within normal ranges and that antifungal drugs were absent. Clinical isolates of *C. neoformans* were cultured in 48-well plates containing Sabouraud dextrose broth (SDB) and incubated at 37°C for 3–4 days until saturation. The cultures were then diluted and inoculated into CSF at a final concentration of 1 ~ 2 × 10^6^ cells/mL, followed by incubation at 37°C for 96 hours. At specified time points (0, 12, 24, 36, 72 and 96 hours post-inoculation), aliquots were collected and viable cells were quantified by plating on Sabouraud dextrose agar (SDA) for CFU counts. The survival slope, representing the mean rate of change in cryptococcal counts over time, was calculated by averaging the slope of the linear regression of log_10_ CFU/ml across time points for each strain.

### Virulence studies

For the *G. mellonella* model of cryptococcosis, larvae were incubated at 30°C for 48 hours. Larvae in poor condition were excluded, and groups of 20 healthy larvae were placed into 10 cm petri dishes. Each larva was injected with 10 μL of a yeast suspension containing 1 × 10^6^ cells into the first segment of the left proleg. Survival status was monitored daily, and survival curves were generated using GraphPad Prism.

For mouse model of cryptococcosis, a well-characterized murine inhalation model of cryptococcosis was employed. Briefly, three isolates were randomly picked from each of the three groups and individually grown overnight in liquid YPD cultures at 30°C. All isolates tested were ST5, except for one environmental strain (Env #103; ST39). Yeast cells were counted using a hemocytometer, and suspensions were prepared at a final concentration of 1 × 10^7^ cells/mL. Female C57BL/6 mice, aged 6–8 weeks, were anesthetized via intraperitoneal injection of ketamine (75 mg/kg) and medetomidine (0.5–1.0 mg/kg). Each mouse was intranasally inoculated with 50 μL of the yeast suspension, containing 1 × 10^5^ cells. Mice were monitored several times per week for signs of disease (weight loss, ruffled fur, shallow breathing, abnormal gait, and lethargy) and then observed daily as symptoms progressed. Mice exhibiting signs of severe morbidity, including significant weight loss, abnormal gait, hunched posture, or cranial swelling, were humanely sacrificed by CO_2_ inhalation followed by cervical dislocation. Survival curves were analyzed using the Kaplan-Meier method in GraphPad Prism 6.0, and differences in median survival among groups were evaluated using the log-rank test.

### Whole genome sequencing

A total of 105 *C. neoformans* isolates, including strains from HIV-I patients, HIV-U patients, and environmental samples (ENV), were selected for whole-genome resequencing. Next-generation sequencing libraries were prepared following the manufacturer’s protocol (NEBNext® Ultra™ DNA Library Prep Kit for Illumina®). For each sample, 1 µg of genomic DNA was randomly fragmented to < 500 bp using a Covaris S220 sonicator. The fragmented DNA was treated with End Prep Enzyme Mix for end repair, 5’ Phosphorylation and dA-tailing in one reaction, followed by a T-A ligation to add adaptors to both ends. Adaptor-ligated DNA was size-selected using AxyPrep Mag PCR Clean-up (Axygen), and fragments of ~ 410 bp (with the approximate insert size of 350 bp) were recovered. Each sample was then amplified by PCR for 8 cycles using P5 and P7 primers, where both primers carried sequences for annealing to the flow cell for bridge PCR, and the P7 primer included a six-base index for multiplexing. The amplified PCR products were purified using AxyPrep Mag PCR Clean-up (Axygen), validated with an Agilent 2100 Bioanalyzer (Agilent Technologies, Palo Alto, CA, USA), and quantified using a Qubit 2.0 Fluorometer (Invitrogen, Carlsbad, CA, USA). Whole-genome sequencing of all strains was performed on an Illumina NovaSeq6000 platform with paired-end 150 bp reads (PE150) at Beijing Novogene Bioinformatics Technology Co., Ltd.

### Data analysis

The raw sequencing data was preprocessed using fastp (v0.23.4) to remove adapter sequences, PCR primers, reads containing more than 10% N bases, and reads with a base quality score below 20. Clean reads were then aligned to the *C. neoformans* H99 reference genome using BWA mem (v0.7.18). The resulting alignment files were processed using Samtools (v1.20) to convert, sort, and index the data. Duplicate reads were marked and removed using GATK’s MarkDuplicates tool. Variant calling, including single nucleotide polymorphisms (SNPs) and indels, was performed using GATK HaplotypeCaller in GVCF mode. Finally, the variants were genotyped using GATK GenotypeGVCFs and annotated with Snpeff (v 5.2a). GO-term enrichment analysis of biological processes was performed for gene sets specific to the HIV-i and HIV-u groups using a locally constructed GO enrichment database, analyzed with the clusterProfiler package. Prior to conducting the genome-wide association study (GWAS), GVCF files from each group pair were merged using GATK CombineGVCF, consolidating variants across groups into a single file. The combined GVCF file was then processed through GATK GenotypeGVCFs for variant calling. Genome-wide mutation distributions were visualized using Circos plots. Variants, including SNPs and indels, and mutation densities were calculated in 50 kb sliding windows across the genome for each group.

### Phylogenetic analysis

For the phylogenetic analysis, variant calls were used to construct a phylogenetic tree. ModelTest-NG was employed to determine the best-fit nucleotide substitution model based on the alignment. A maximum likelihood phylogenetic tree was constructed using IQ-TREE (v2.4) based on a multiple sequence alignment. The best-fit substitution model (GTR+G4) was selected according to the Bayesian Information Criterion using ModelTest-NG. The resulting phylogeny was visualized using ggtree in R (v4.4.0), with additional phenotype annotations implemented via ggtreeExtra and ggnewscale. To assess population structure, a principal component analysis (PCA) was conducted on the variant data obtained from GATK output using PLINK. The PCA results were visualized using ggplot2 in R (v4.4.0), revealing population structure across the isolates.

### GWAS analysis

Prior to running the GWAS, a rigorous variant filtering step was performed to ensure high quality of variant calls. Variants were first filtered using Bcftools based on quality metrics such as read depth, mapping quality, and strand bias (e.g. QUAL ≥ 30, MQ ≥ 40), retaining only high-confidence variants for analysis. Additionally, a second filtering step targeted functional annotations, selecting variants predicted to cause significant changes in protein function, including missense, nonsense, frameshift, and splice site variants. This two-step filtering process refined the variant dataset, improving the reliability and biological relevance of downstream GWAS results. The GWAS analyzed three phenotypes: polysaccharide capsule size, survival time post-*Galleria mellonella* infection, and melanin production. Variant data were processed using PLINK, converting the VCF file into binary format and generating a relatedness matrix for the isolates. Association testing was performed using the linear mixed model (LMM) implemented in GEMMA, which accounted for genetic relatedness to minimize confounding effects. To visualize the distribution of *p*-values from the GWAS, a quantile–quantile (QQ) plot was generated. Observed *p*-values were sorted, transformed using a -log_10_ scale, and plotted against expected *p*-values under a uniform distribution. The plot was created using a custom Python script with Matplotlib. After GWAS, filtering criteria were applied to identify significant mutations for further analysis. For each phenotype – survival post-*G. mellonella* infection, melanin production, and polysaccharide capsule size – variants were filtered based on significance thresholds for the -log_10_ (*p*-value), set at 2. This approach yielded tens of relevant mutations per phenotype, enabling focused downstream functional validation. A Manhattan plot was generated to visualize the genome-wide distribution of variant – phenotype associations across 15 chromosomes, with significantly associated loci labeled by gene names. Variants were plotted by chromosomal position and – log₁₀ (p-value) of association.

### Statistics

Data are presented as mean ± standard error of the mean (SEM). Statistical analyses were performed using one-way analysis of variance (ANOVA) in GraphPad Prism 6.0 software (San Diego, CA). Significance levels were defined as follows: * *p* < 0.05; ** *p* < 0.01; *** *p* < 0.001; **** *p* < 0.0001.

## Results

### Characterization of clinical presentation and outcome

To investigate the correlations amongst environmental conditions, immune inflammatory syndrome and other clinical outcomes, we analyzed 98 clinical isolates of *C. neoformans* collected from hospitalized patients across different locations in China. These isolates were recovered from cerebrospinal fluid (CSF) samples (*n* = 90) and blood cultures (*n* = 8). Among the isolates, 54 originated from HIV-infected patients, while 44 were from HIV-u individuals. Clinical laboratory data were available for 95 patients (data for three patients were unavailable). Detailed information regarding the influence of symptoms on clinical outcomes is presented in Table 1, highlighting potential relationships between isolate origin and patient outcomes. The cohort comprised predominantly male patients (74/95; 77.9%), with an age range of 21–68 years and a mean age of 41 ± 11 years. Headache emerged as the most common presenting symptom, reported in 70% of patients, with a higher prevalence among HIV-infected individuals (73%). In contrast, HIV-uninfected patients exhibited fewer instances of high fever but experienced more severe neurological complications, including seizures, cerebral herniation, and cerebellar signs. Interestingly, 39 of 51 HIV-infected patients had CD4^+^ T-cell counts below 50/μL, emphasizing their severe immunosuppression. By comparison, the mean ± SD CD4^+^ T-cell count among HIV-uninfected patients was significantly higher at 381 ± 92/μL. These findings align with previous studies, which have demonstrated that HIV-associated cryptococcal meningitis typically occurs in individuals with profoundly low CD4^+^ T-cell counts [34].

**Table 1.** Comparison of clinical data for HIV-uninfected and -infected groups.

		Mean ± SD (*n*)^a^		
		Patients		
Parameter	Total subjects^b^	HIV-u 44 isolates	HIV-i 51 isolates	*p*-value^c^
Age (yr)	41 ± 11	38 ± 9	43 ± 12	0.0679
Sex (males/females)	74/21	33/11	41/10	0.5792
CD4 Count (no. of cells/μL)	193 ± 188	381 ± 92	34 ± 48	<0.001
Temperature (°C)	37.4 ± 0.9	37.1 ± 0.6	37.6 ± 1.1	0.0037
Fever (Y/N)	45/50	12/32	33/18	<0.001
Headache(Y/N)	66/29	29/15	37/14	0.5105
Seizure(Y/N)	24/71	16/28	8/43	0.0207
Cerebral herniation(Y/N)	47/48	12/32	4/47	0.0143
Cerebellar Signs(Y/N)	16/79	28/16	20/31	0.0176

^a^Values, including age, CD4 counts and temperature, are presented by means ± SD for the statistic characteristics and clinical parameters. *n* indicates the number of patients.

^b^Total subjects are reported as the mean ± SD for each group of patients.

^c^*p* values were calculated by one-way ANOVA or Fisher’s exact test as appropriate.

### MLST and phylogenetic analyses

**Figure 1.**
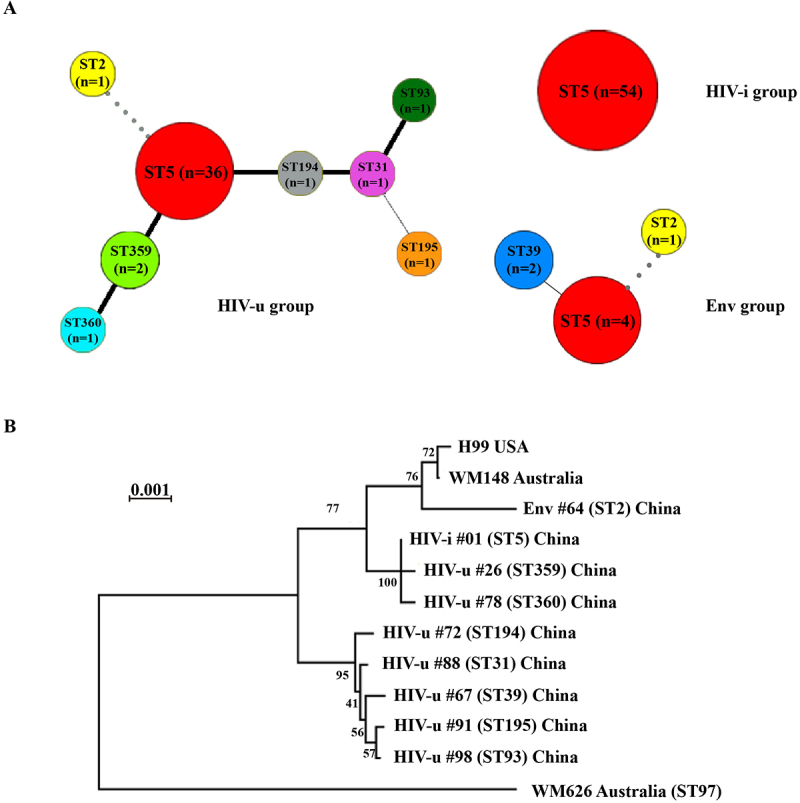
Genetic and evolutionary relationships among *C. neoformans* strains derived from different sources. (A) phylogenetic relationships of 105 *C. neoformans* strains based on the MLST sequencing analysis. Sequence types (STs) are color-coded, with the number of isolates sharing each ST indicated at the respective nodes. Phylogenetic connections between STs are represented by lines, with the line type (bold black, plain black, or grey) reflecting the number of allelic mismatches between profiles: bold black ( < 3 allelic mismatches), plain black (3–4 allelic mismatches), and grey ( > 4 allelic mismatches. (B) phylogenetic tree of selected *C. neoformans* strains constructed from DNA sequences of eight housekeeping gene loci. The tree was generated using the neighbor-joining (NJ) method with 1,000 bootstrap replicates for support. Strains labeled as HIV-u, HIV-i, or Env correspond to isolates from this study’s 105 Chinese isolates. Additionally, three reference strains, one from the United States and two from Australia, are included for comparison.

The 98 clinical isolates, together with 7 environmental strains collected from pigeon excreta, were classified into three groups: the HIV-u group (44 strains), the HIV-i group (54 strains), and the Env group (7 strains). After colony purification, all 105 isolates were genotyped using MLST, following a standardized consensus typing scheme based on seven housekeeping genes (*CAP59*, *GPD1*, *IGS1*, *PLB1*, *LAC1*, *SOD1*, *URA5*) [30]. For each strain, locus allele identifiers were used and combined to generate a sequence type (ST), representing the strain genotype [35]. MLST analysis revealed 9 distinct sequence types (STs) among the 105 isolates, with the majority (94/105; 89.5%) belonging to ST5. The remaining isolates were distributed across ST359 (*n* = 2; 1.9%), ST2 (*n* = 2; 1.9%), ST39 (*n* = 2; 1.9%), ST360 (*n* = 1; 0.9%), ST194 (*n* = 1; 0.9%), ST31 (*n* = 1; 0.9%), ST93 (*n* = 1; 0.9%), and ST195 (*n* = 1; 0.9%) (Figure 1A). Notably, all isolates from the HIV-i group were exclusively of a single sequence type (ST5), as detailed in Table S1. In contrast, isolates from the HIV-u and Env groups displayed greater diversity: the HIV-u group harbored eight STs, including ST5 (*n* = 36), ST359 (*n* = 2), ST2 (*n* = 1), ST360 (*n* = 1), ST194 (*n* = 1), ST31 (*n* = 1), ST93 (*n* = 1), and ST195 (*n* = 1). Similarly, the Env group included three STs: ST5 (*n* = 4), ST39 (*n* = 2), and ST2 (*n* = 1). Phylogenetic analysis, based on the concatenated sequences of the seven MLST loci, was performed to investigate the relationships among isolates from different geographic locations (China, Australia, Brazil, and the USA) (Figure 1B). The resulting phylogenetic tree demonstrated that ST5 was closely related to ST359 and ST360, whereas ST39 appeared phylogenetically distant from the other identified STs. These findings corroborate previous studies, which identified ST5 as the dominant epidemic clone of *C. neoformans* var. *grubii* in China, particularly among isolates from HIV-uninfected individuals [36,37].

As shown in Figure 1A, the predominance of ST5 was evident across all groups, accounting for 54/54 isolates in the HIV-i group, 36/44 isolates in the HIV-u group, and 4/7 isolates in the Env group. This striking distribution of ST5 prompted further investigation into the potential relevance of isolate origins, beyond sequence type, in determining virulence characteristics.

### *In vitro* and *ex vivo* phenotyping

*C. neoformans* is known for producing several key virulence factors, most notably the mating type, polysaccharide capsule, and melanin production, each of which contributes to its pathogenic potential. Previous studies have shown that mating type plays a critical role in virulence, influencing cell type (*MATµ* or *MAT*a) and activity of specific genes particularly those involved in MAP kinase signaling pathway [38–40]. To investigate the mating types and serotypes of the clinical isolates, we performed multiplex PCR using the primers previously described [30]. Among the 105 strains analyzed, 104 were characterized as serotype A *MAT*α (Aα), and only one strain was found to be serotype A *MAT*a (Aa) (Figure S1).

**Figure 2.**
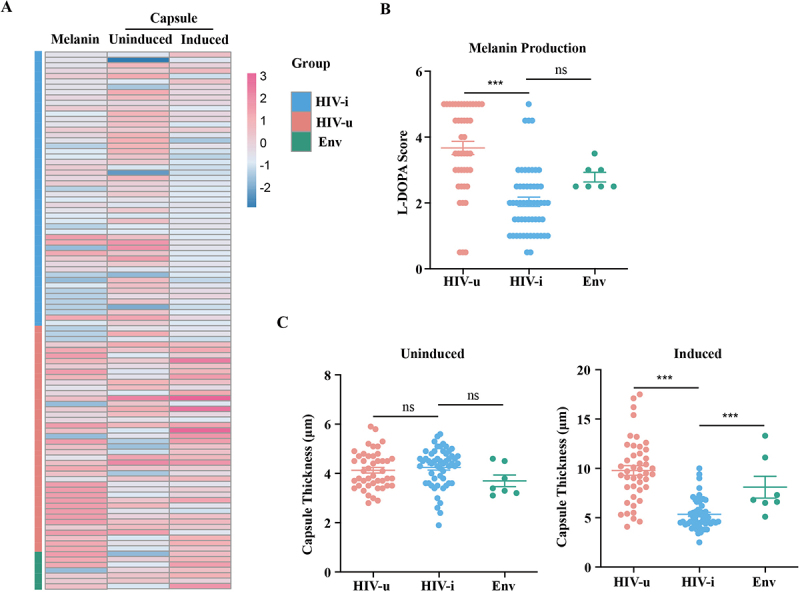
*In vitro* characterization of melanin biosynthesis and polysaccharide capsule formation in *C. neoformans* isolates from each of the three distinct groups. (A) The heatmap was organized into columns for strains and rows for phenotypes. Red and blue indicate high and low scores to phenotypes, respectively. The data are presented as individual values from two biological replicates. (B) Statistical comparison of melanin biosynthesis across all 105 of *C. neoformans* isolates. Melanin production was categorized using *K*-means cluster analysis, with vertical bars indicating standard errors of the mean for each group. Statistical significance is denoted as. ns, no significance, *p >* 0.05, *** *p* < 0.001. (C) Statistical comparison of capsule formation in all 105 *C. neoformans* isolates. Fungal cultures at stationary phase were washed, resuspended in PBS, and diluted 1/100 in capsule-inducing medium (10% Sabouraud dextrose in MOPS buffer at pH 7.3). After incubation at 30 °C and 180rpm for 48 hours, India ink-stained suspensions were photographed, and capsule thickness of each isolate was measured. Each symbol represents the average capsule thickness was measured for each isolate. Each symbol represents the average capsule thickness of 10–20 cells. Statistical significance is indicated by *p < 0.05, ***p < 0.001, determined by unpaired two-tailed Student’s t-test.

Melanin production is another major virulence factor in *C. neoformans*. To assess the ability of the 105 clinical isolates to produce melanin, we performed *in vitro* assays by culturing the isolates on medium containing L-DOPA (L-3,4-dihydroxyphenylalanine) [41]. A scoring method based on K-means clustering analysis was employed to evaluate the extent of melanin production across all tested strains. Two reference strains, (JEC20 and H99), were included as experimental controls. Strain JEC20 is known for producing minimal melanin, while the clinical isolate H99 exhibits robust melanization [42]. As shown in Figure 2A,B and S2, strains from the HIV-u group exhibited significantly higher levels of melanin, as evidenced by the darker pigmentation and higher scores compared to those from the HIV-i group (*p* < 0.0005 by Tukey-adjusted t-test). These results suggest that the clinical isolates from the HIV-u group may exhibit distinct differences in pathogenicity compared to those from the HIV-i group.

This notion was further supported by assaying the polysaccharide capsule formation of each isolate. We observed that after induction, strains in HIV-u group generate much larger capsules than those in HIV-i group, although their sizes are almost indistinguishable under un-induced condition (Figure 2A,C, S3, S4 and S5).

**Figure 3.**
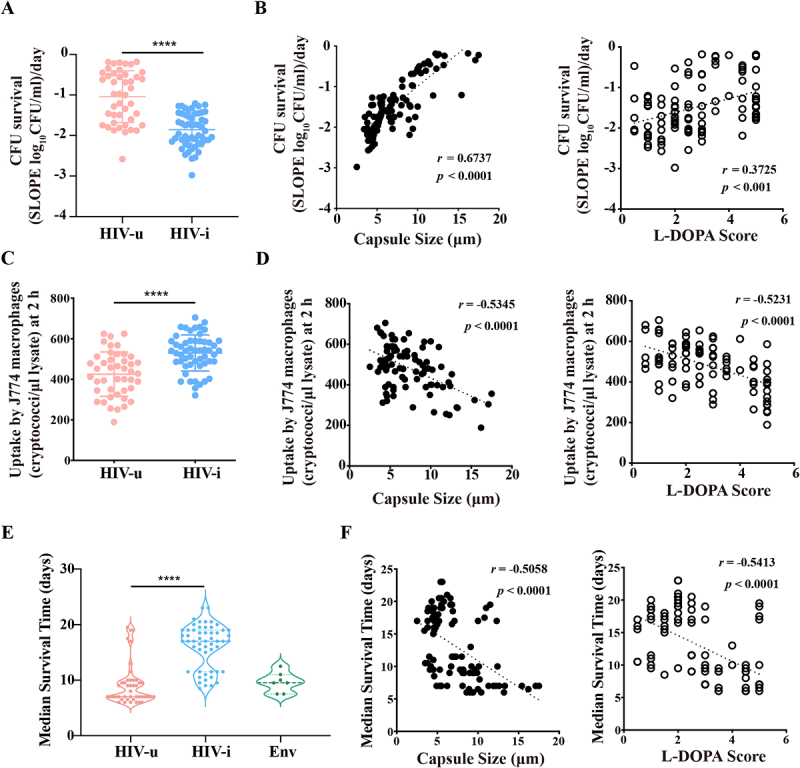
*Ex vivo* and *in vivo* assessment of virulence-associated traits in *C. neoformans* isolates from three distinct groups. (A, B) Cryptococcal survival in human CSF. Clinical isolates were inoculated into human CSF at a concentration of 1 ~ 2 × 10^6^ cells/ml, and aliquots were collected at different time points (0, 12, 24, 36, 72 and 96 hours post-inoculation). Samples were plated on Sabouraud dextrose agar (SDA) media to quantify colony-forming units (CFUs). The survival slope was calculated as the mean rate of change in CFU/mL over time, determined by averaging the slope of the linear regression of log_10_ CFU/mL for each strain A. Survival comparison of clinical *C. neoformans* strains between the HIV-u and HIV-i groups after incubation with human cerebrospinal fluid (CSF); B. Correlation between capsule size or L-DOPA score and *ex vivo* cryptococcal survival in human CSF. (C, D) Phagocytic uptake of clinical *C. neoformans* strains by the macrophage-like cell line J774. Macrophage-like cells (1.5 × 10^5^) were incubated in serum-free DMEM medium for 2 hours, followed by activation with 15 µg/mL phorbol myristate acetate (PMA) for 30–60 minutes. Subsequently, the cells were co-incubated with *C. neoformans* yeast cells that had been pre-opsonized with a monoclonal antibody for 2 hours at 37°C with 5% CO₂ (MOI = 1:10). Phagocytosis was assessed by quantifying the number of cryptococci internalized by macrophages 2 hours post-infection. Data are presented as the mean of 3–4 independent experimental repeats. C. Phagocytic uptake comparison of clinical *C. neoforman*s strains between HIV-u and HIV-i groups after incubation with J774 macrophage-like cells D. Association of capsule size or L-DOPA score with *ex vivo* phagocytic uptake. (E) The median survival time of *G. mellonella* infected with isolates from each of the three groups. The *G. mellonella* (*n* = 20) were infected with 5 × 10^5^ CFUs of individual strains and monitored for progression to severe morbidity. (F) Association of capsule size or L-DOPA score with median survival time of *G. mellonella*.

The ability of *C. neoformans* to survive in cerebrospinal fluid (CSF) is an important factor in its virulence, as it is crucial for the organism’s presence in clinical specimens and its ability to cause life-threatening central nervous system (CNS) infections. To evaluate the pathogenic differences between isolates from HIV-i and HIV-u individuals, we tested the survival of all 98 clinical isolates in human CSF. Strains from the HIV-u group demonstrated greater resistance to CSF-mediated killing compared to those from the HIV-i group (Figure 3A). Additionally, a strong positive correlation was found between CSF survival and capsule size (*r* = 0.6737, *p* < 0.0001) or L-DOPA score (*r* = 0.3725, *p* < 0.001; Figure 3B), further suggesting that the larger capsule size and more melanin production in the HIV-u group contributes to enhanced survival in the CSF. These findings reinforce the hypothesis that strains from HIV-uninfected individuals exhibit higher virulence than those from HIV-infected patients.

Furthermore, macrophage internalization is a critical virulence factor in *C. neoformans* infections, with several studies highlighting its importance in pathogenesis [43,44]. Interestingly, our analysis of macrophage uptake in the murine macrophage-like cell line J774 revealed that isolates from the HIV-i group were phagocytosed at a higher rate than those from the HIV-u group (Figure 3C). This phenomenon could be attributed to differences in capsule size and melanin production, as we observed an inverse correlation between phagocytic uptake and capsule size (*r* = −0.5345, *p* < 0.0001) or melanin production (*r* = −0.5231, *p* < 0.0001; Figure 3D). These results are consistent with previous studies demonstrating that the capsule and melanization impedes phagocytosis of *C. neoformans* by macrophages, and that capsule-dependent anti-phagocytic activity is a major virulence factor [45,46].

Taken together, both *in vitro* and *ex vivo* assays indicate that isolates from HIV-uninfected individuals exhibit significantly enhanced capsule production and melanin formation, increased survival in CSF, and reduced phagocytic uptake, all of which are key factors contributing to the virulence of *C. neoformans*. These findings highlight the greater pathogenic potential of *C. neoformans* strains from HIV-uninfected individuals compared to those from HIV-infected patients.

### *In vivo* virulence analyses

The observed differences in capsule and melanin production between *C. neoformans* strains from HIV-uninfected and HIV-infected individuals prompted us to explore whether the origin of the strain affects its virulence. To address this, we conducted an *in vivo* virulence study using the *G. mellonella* larvae model system [47]. The *G. mellonella* larvae were inoculated with 105 strains from the three groups, and their survival rate was monitored for 21 days. Strikingly, larvae infected with HIV-u strains exhibited near-complete mortality, with a median survival time of 9.1 days. In contrast, larvae infected with HIV-i strains exhibited 61.1% survival at day 21, with a median survival time of 16.2 days (Figure 3E, S6). These results clearly show that strains from HIV-u individuals are significantly more virulent than those from both HIV-i and Env groups. Additionally, a strong negative correlation was found between median survival time of *G. mellonella* and capsule size (*r* = −0.5058, *p* < 0.0001) or L-DOPA score (*r* = −0.5413, *p* < 0.001; Figure 3F), indicating that the capsule and melaninization contribute to the virulence of *C. neoformans* in *G. mellonella*. We also conducted an *in vivo* virulence assay using a mouse model of cryptococcosis to further assess the pathogenicity of the strains [48]. Groups of 6–8-week female C57BL/6 mice (8 per group) were intranasally inoculated with strains from the three study groups, with three randomly picked strains from each group, alongside the H99 strain serving as a control. All isolates tested were ST5, except for one environmental strain (Env #103; ST39). Mice were euthanized by CO_2_ inhalation followed by cervical dislocation upon displaying signs of severe morbidity, including weight loss, abnormal gait, hunched posture, and cranial swelling. Consistent with previous studies [48], the control strain H99 caused a lethal infection within 20 days. As expected, mice infected with HIV-u strains exhibited similar or even worse symptoms than those infected with the control strain, while mice infected with HIV-i and Env strains demonstrated significantly better survival rates (Figure S7). These strains were much less virulent than those from the HIV-u group.

Taken together, these *in vivo* findings corroborate our *in vitro* and *ex vivo* results, demonstrating that strains of *C. neoformans* from different host groups exhibit distinct virulence profiles. This suggests that environmental factors, including the host’s immune status, significantly influence the evolution of *C. neoformans* virulence.

### Population genomic analysis reveals potential genetic association among the groups

Microbial pathogens are known to evolve a broad range of intrinsic and extrinsic strategies that enable them to acquire and modulate virulence traits, thereby facilitating successful host colonization [49]. While environmental factors play a significant role, the pathogenicity of *C. neoformans* is also deeply influenced by genetic variation, a fundamental aspect of microbial life. This genetic diversity allows pathogens to adapt to their hosts and evade immune defenses. Furthermore, whole-genome resequencing has proven to be an invaluable tool for exploring the genetic mechanisms underlying fungal-host interactions [50].

In this study, we sequenced and assembled the whole genomes of 105 *C. neoformans* strains, including 7 from the ENV group, 44 from the HIV-u group, and 54 from the HIV-i group. We aimed to uncover potential genetic correlations with virulence by comparing the genome differences among the 105 isolates against the reference strain H99. Quality control analyses of our sequencing data are summarized in Table S3. In total, the sequencing effort generated 448.74 Gb of raw data, containing three billion reads, with an average of 28.5 million reads per sample. After filtering out low-quality and duplicate reads, we obtained 438.33 Gb of high-quality data, totaling 2.93 billion reads. Six samples, with alignment rates below 40%, were excluded to ensure the reliability of the analysis, reducing the potential impact of low-quality or contaminated data. Approximate 92.5% of the clean reads could be mapped to the H99 reference genome (Table S4). Using GATK and SnpEff, we conducted mutation analysis on the aligned genomes, identifying a total of 553,671 variants. Most samples exhibited mutation counts within a consistent range, while 7 samples displayed abnormally high or low mutation frequencies. The number of mutations observed in the 105 clinical isolates, compared to the H99 reference genome, is summarized in Table S5.

To examine evolutionary relationships across the isolates, we further constructed a phylogenetic tree based on the mutation sites in the VCF files, excluding the 13 abnormal samples. The phylogenetic analysis revealed that while a subset of the HIV-i isolates clustered into two distinct genetic clades exhibiting higher virulence including high scores of melanin and capsule and low survival scores, with one HIV-u isolate found in each clade (pink labeled), the majority of strains from different clinical and environmental sources did not form clear genetic clusters (Figure S8A). This suggests that there may be potential genetic exchange and strain transmission among the three ecological niches. This hypothesis was further supported by principal component analysis (PCA) of sequence similarity (Figure S8B), which indicated possible genetic communication between strains from different environments.

Next, we conducted a comparative analysis of mutation site counts across the three groups, HIV-u, ENV, and HIV-i. Figure S9A shows the relationship between the number of variants and genes, with an inset that highlights genes containing at least 10 variants in the HIV-u and HIV-i groups, and at least five variants in the ENV group. The analysis revealed no significant differences between the groups. Figure S9B demonstrates that the majority of genes across all three groups harbor relatively few variants, with no correlation between gene length and variant number. This suggests that the genetic variation in these isolates is relatively uniform and independent of their origin. These variants were distributed across the genome as shown in Figure S9C, with the top 10 genes harboring the most variants labeled for each group. To analyze the genomic distribution of various mutation types across the three groups, we performed a Circus plot displaying insertions, deletions, synonymous SNPs, and non-synonymous SNPs. Notably, the density of synonymous and non-synonymous mutations is higher than that of insertions and deletions (Figure S10). The distribution of various mutation types differs substantially across genomic regions, reflecting clear chromosomal specificity, with mutations more frequently observed in telomeric regions. The HIV-i and HIV-u groups exhibit similar mutation patterns across several chromosomes; however, in certain regions (e.g. NC_026751.1 and NC_026750.1), the HIV-u group shows a markedly higher mutation frequency. This, combined with the more polyphyletic branching observed in the co-evolutionary tree (Figure S8A), may be related to differences in fungal virulence or transmission potential. In contrast, the ENV group exhibits relatively fewer mutations overall, especially in the synonymous and non-synonymous categories, indicating lower mutational pressure. Some genomic regions (e.g. NC_026755.1, NC_026749.1, and NC_026756.1) display consistently high mutation levels across all groups, potentially representing hotspots for structural variation or regions enriched in functional genes.

### Isolates from HIV-u group are enriched for carbohydrate metabolism relative to HIV-i group

**Figure 4.**
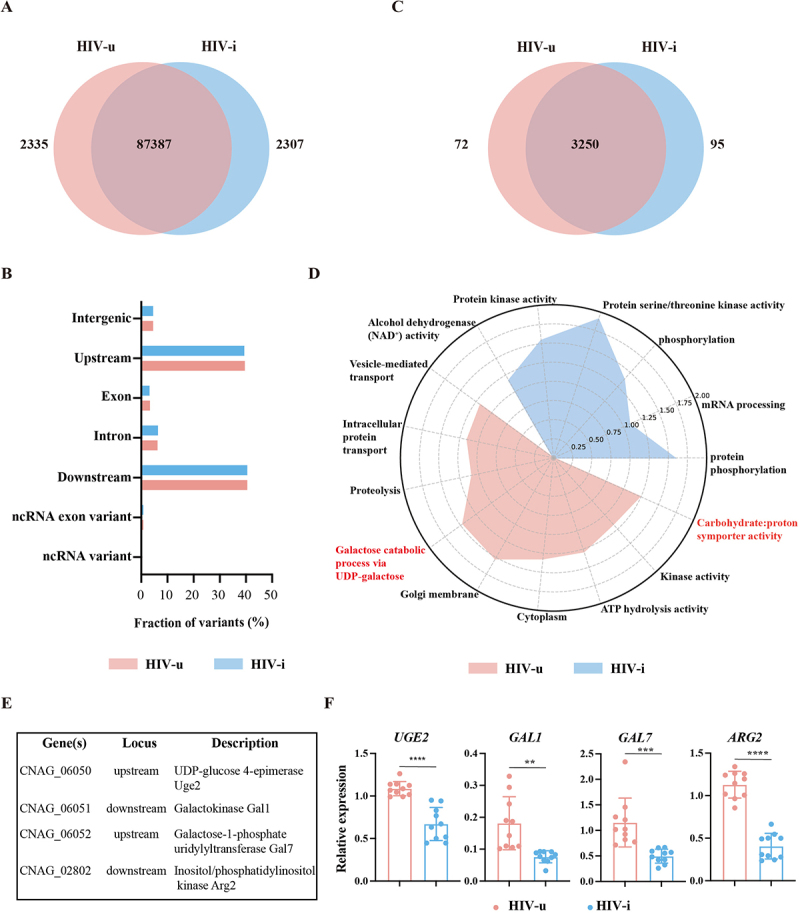
Common and group-specific variants and genes identified in *C. neoformans* isolates from HIV-u and HIV-i groups. (A) venn diagram illustrating the distribution of common and group-specific variants in *C. neoformans* isolates. A common variant is defined as one found in more than 50% of isolates from each group. (B) genomic distribution of group-specific variants. (C) venn diagram showing the number of genes associated with group-specific variants. Of note, 3,250 genes are common to both groups, though variants in these genes were mapped to different positions within each group. (D) radar chart displaying enriched gene ontology (GO) terms for *C. neoformans* isolates from HIV-u and HIV-i groups. The chart presents significantly enriched GO terms related to biological processes, cellular components, and molecular functions. The radial axis indicates the – log_10_ (*p*-value), with higher values reflecting stronger statistical significance of the enrichment. (E) Table listing indicated genes with gene IDs, loci, and functional descriptions. (F) expression levels of Uge2, Gal1, Gal7 and Arg2, as determined by real-time RT-qPCR, in *C. neoformans* isolates from the HIV-i and HIV-u groups under YPD condition (*n* = 10 biologically independent samples). Gene expression data were normalized to the control gene GAPDH and are presented relative to the negative control, which was set to 1. Data are expressed as the mean ± SD, and are representative of three independent experiments. **p < 0.01, ***p < 0.001, ****p < 0.0001, by Student’s t-test.

To investigate whether the observed phenotypic traits in each group are associated with specific genetic variants, we conducted an extensive genome comparison between the H99 reference strain and clinical isolates from the HIV-u and HIV-i groups. This analysis identified polymorphisms unique to each group. As shown in Figure 4A, we found 87,387 common SNPs (defined as variants present in more than 50% of isolates) across both groups, along with 2,335 and 2,307 variants specific to the HIV-u and HIV-i groups, respectively. Moreover, no significant differences in variant distribution were observed between the two groups in gene regions such as intronic, downstream, and ncRNA_exonic regions (Figure 4B). Further investigation revealed that the 2,335 unique variants in the HIV-u isolates were associated with 72 genes, while the 2,307 unique variants in the HIV-i isolates were linked to 95 genes (Figure 4C).

Interestingly, functional enrichment analysis, depicted in a radar plot, revealed significant differences in gene functions between *C. neoformans* strains isolated from HIV-i and HIV-u patients. HIV-u strains were enriched in biological functions closely related to carbohydrate transport and metabolism, ATP hydrolysis activity, vesicle-mediated or intracellular protein transport, functions known to be important for *C. neoformans* pathogenicity. In contrast, HIV-i strains exhibited enrichment in mRNA processing and protein phosphorylation (Figure 4D). A list of the major mutated genes and their associated functions is provided in Table S6. Notably, isolates from the HIV-u group harbored specific variants in the genes *UGE2* (*CNAG_06050*), *GAL1* (*CNAG_06051*), *GAL7* (*CNAG_06052*) and *ARG2* (*CNAG_02802*), which are key players in utilizing galactose as a carbon source. These genes are also reported to contain a conserved PDKG motif, essential for the catalytic activity of IP3 kinases, and may play an important role in the inositol polyphosphate anabolic pathway, which is essential for virulence of *C. neoformans* [51–53]. *CNAG_00312* (synaptobrevins) and *CNAG_04074* (coatomer beta’ subunit), which are enriched in the vesicle-mediated transport pathway, may promote the transport of glucuronoxylomannan, a major polysaccharide component of the *C. neoformans* cell wall capsule, and further contribute to capsule secretion or melanin deposition [54]. In the HIV-i group, we identified several kinases (*CNAG_03670*, *CNAG_05216* and *CNAG_05439*) carrying group-specific mutations, which are involved in protein kinase activity and phosphorylation processes. This suggests that these strains may have significant differences in signal transduction, metabolic regulation, or stress response. In addition, we found a gene, *CNAG_00111*, which encodes splicing factor 3A subunit 1 and may be involved in mRNA splicing, potentially affecting the gene expression.

Given the important role of carbohydrate metabolism-related genes in inositol anabolic of *C. neoformans* and mutation position of these genes (Figure 4E), we hypothesized that these genes are highly expressed in HIV-u strains. To verified our hypothesis, we randomly selected ten representative isolates from each group and compared the expression of these genes between isolates from HIV-u and HIV-i groups. Consistent with our predictions, the expression levels of *UGE2*, *GAL1, GAL7* and *ARG2* were higher in HIV-u strains compared to HIV-i strains (Figure 4F).

These data strongly suggest that compared *C. neoformans* strains isolated from HIV-infected patients, those from HIV-uninfected individuals have evolved specific genetic variations in genes related to environmental adaptation, possibly due to the more challenging host environment they face, including immune system clearance.

### GWAS analysis identifies key genetic associations with virulence-related traits

**Figure 5.**
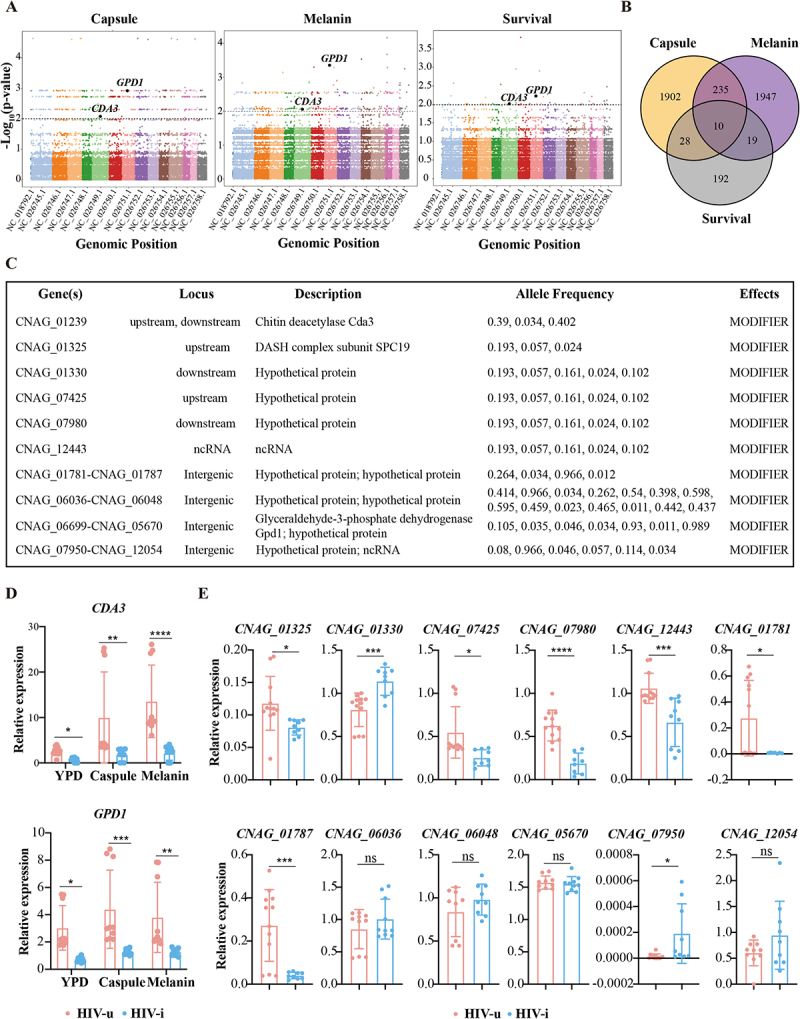
Analysis of gene associations with virulence factors (capsule formation, melanin production, and survival) in *C. neoformans* isolates from HIV-u and HIV-i groups. (A) The Manhattan plot illustrates the distribution of genetic variants across 15 chromosomes. Each dot represents a single variant, plotted according to its chromosomal position (x-axis) and the – log₁₀ (p-value) of its association with the phenotype (y-axis). Chromosomes are displayed in alternating colors for visual clarity. Labeled points indicate variants that show significant association with the phenotype; the interest genes are indicated in black. (B) Venn diagram showing the overlap of genes associated with capsule formation, melanin production, and survival. Each circle represents a set of genes associated with one trait, with overlapping regions indicating genes that may play roles in multiple virulence factors. Notably, 10 genes are shared among all three traits, suggesting potential multifunctional roles. (C) Table listing shared genes among capsule formation, melanin production, and survival traits, with gene IDs, loci, allele frequency, predicted effects annotated by Snpeff and functional descriptions. (D) qRT-PCR analysis of *CDA3* and *GPD1* in HIV-u and HIV-i strains grown under YPD conditions. *CDA3* and *GPD1* are functionally linked to carbohydrate metabolism pathways (e.g. carbohydrate: proton symporter activity) as highlighted in Figure 4D. Transcript levels were normalized to the level of *GADPH* mRNA. Results from three independent experiments are shown. (E) qRT-PCR analysis of the indicated genes in HIV-u and HIV-i strains grown under YPD conditions. Transcript levels were normalized to the level of *GADPH* mRNA. Results from three independent experiments are shown. All data are expressed as means ± SD. ns, no significance, *p < 0.05; **p < 0.01, ***p < 0.001, ****p < 0.0001, by Student’s t-test.

To investigate the genetic determinants of virulence in *C. neoformans*, we conducted a genome-wide association study (GWAS) targeting three critical phenotypic traits: melanin production, capsule formation, and survival time post-infection with *Galleria mellonella*. The Manhattan plots and Q-Q plots for the GWAS analysis of melanin, capsule, and survival phenotypes were performed to showed the distribution of significant phenotype-related mutations on the genome and to reduced false positives and obtain more significant SNPs (Figure 5A and S11). At a significance threshold of *p* < 0.01, we identified 357 SNPs associated with capsule formation, 374 SNPs linked to melanin production, and 51 SNPs related to survival time, which mapped to 1684, 1381, and 188 gene regions, respectively. Strikingly, a subset of genes associated with all three traits was identified, highlighting their potential roles in fungal virulence (Figure 5B). Interestingly, within 50 kb genomic windows in Circos plots (Figure S10), the mutation frequencies of most of these genes were significantly higher in the HIV-u group compared to the HIV-i group. This difference was particularly notable in the window containing *CDA3*, where the number of synonymous mutations reached 2,100 in the HIV-u group, in contrast to only 620 in the HIV-i group.

Among these genes, chitin deacetylase (*CDA3*, *CNAG_01239*) and glyceraldehyde-3-phosphate dehydrogenase (*GPD1*, *CNAG*_*06699*) stand out due to their well-documented involvement in fungal virulence (Figure 5C), which identified in our GWAS, are functionally linked to carbohydrate metabolism pathways highlighted in Figure 4D and Table S6 (carbohydrate: proton symporter activity and galactose catabolic process via UDP-galactose), supporting their role in host-specific virulence adaptation. The chitin deacetylase gene *CDA3* encodes an enzyme involved in the modification and remodeling of the fungal cell wall and the glycolytic enzyme *GPD1* involved in carbohydrate metabolism, has been linked to virulence through its role in host-pathogen interactions [55–57]. Based on the mutation position of these genes (Figure 5C), we randomly selected ten representative isolates from each group and evaluated the expression of these genes in the HIV-u and HIV-i groups. Interestingly, both *CDA3* and *GPD1* were significantly more highly expressed in HIV-u strains compared to HIV-i strains under both standard culture (YPD) conditions and virulence-inducing (capsule or melanin production) conditions, with even more pronounced under the stress condition (Figure 5D), suggesting a potential role for *CDA3* and *GPD1* in contributing to the enhanced virulence or environmental adaptability of HIV-u strains. Moreover, we investigated the expression of hypothetical proteins with unknown functions between the two groups. Several genes were downregulated in HIV-i isolates, including *CNAG_01325*, *CNAG_07425*, *CNAG_07980*, *CNAG_12443*, *CNAG_01781* and *CNAG_01787*), suggesting that these genes might promote virulence in *C. neoformans*. Conversely, the upregulation of *CNAG_01330* and *CNAG_07950* in HIV-i strains implies that these genes might play a suppressive role in virulence. In particular, other genes such as *CNAG_06036*, *CNAG_05670*, and *CNAG_12054* did not show significant expression differences between the HIV-u and HIV-i groups (Figure 5E), suggesting that they may not be involved in the differential virulence observed between the two groups. However, the exact roles of these genes await further investigation.

**Figure 6.**
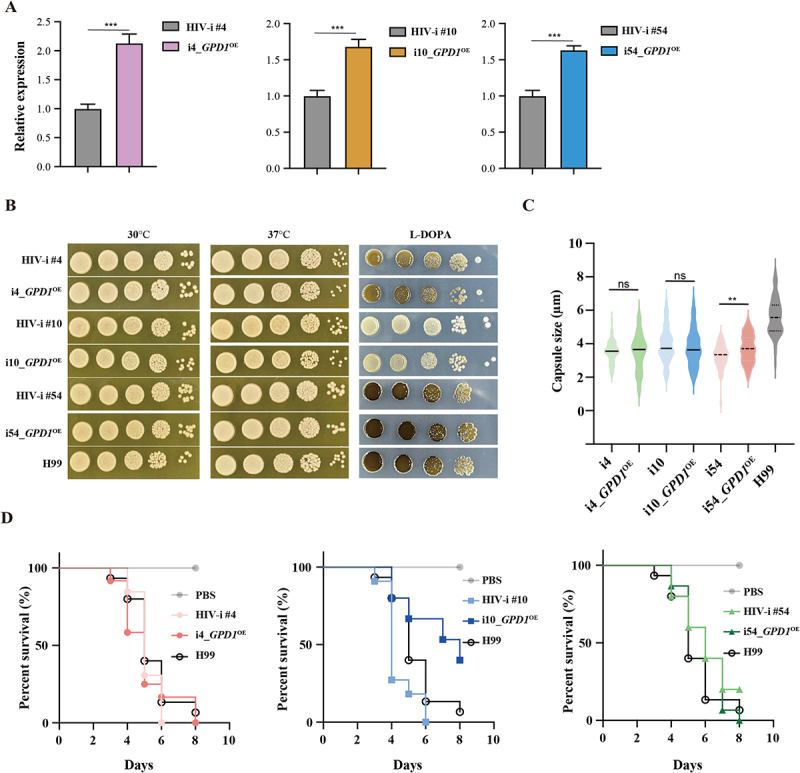
Overexpression of *GPD1* contributed to virulence-related phenotypes of HIV-i strains. (A) expression of *GPD1* in indicated strains under YPD condition (*n* = 3 biologically independent samples). Gene expression data were normalized to the control gene GAPDH and are presented relative to the negative control, which was set to 1. (B) growth of indicated *C. neoformans* strains were spotted with tenfold serial dilutions onto YPD or L-DOPA plate and grown for 2 d at 30°C or 37°C. (C) statistical comparison of capsule formation in indicated *C. neoformans* strains. Fungal cultures at stationary phase were washed, resuspended in PBS, and diluted 1/100 in capsule-inducing medium (10% Sabouraud dextrose in MOPS buffer at pH 7.3). After incubation at 30 °C and 180rpm for 48 hours, India ink-stained suspensions were photographed, and capsule thickness of each isolate was measured. Each symbol represents the average capsule thickness was measured for each isolate. Each symbol represents the average capsule thickness of 10–20 cells. (D) survival curves of *G. mellonella* infected with indicated *C. neoformans* strains. The *G. mellonella* (*n* = 20) were infected with 1 × 10^6^ CFUs of indicated strains and observed for signs of severe morbidity, and survival was tracked over time. Data are expressed as the mean ± SD, and are representative of three independent experiments. **p < 0.01, ***p < 0.001, by Student’s t-test (A, C) and log-rank test (D).

To confirm whether the highly expression of *CDA3* and *GPD1* contributes to virulence-related phenotypes, we overexpressed *GPD1* in three representative HIV-i isolates (repeated attempts to overexpress the *CDA3* gene failed, Figure 6A). The overexpression of *GPD1* showed a slight enhancement in melanin production in the HIV-i4, i10 and i54 strains compared to their parental strains, as well as a modest increase in capsule formation in HIV-i54. However, these modifications did not reach the levels observed in the H99 strain (Figure 6B,C). Overall, overexpression of *GPD1* has minimal impact on the virulence phenotypes, including growth at 30°C and 37°C, melanin production, capsule induction, and virulence in *G. mellonella* (Figure 6D). This might be due to the enhanced virulence phenotype of the HIV-u strain is a result of the combined effects of multiple genes rather than the action of a single gene.

In summary, the differential expression of these genes under distinct host conditions strongly suggests that *C. neoformans* may alter its gene expression profile in response to the host immune status of the host. This adaptive strategy likely influences the pathogen’s virulence, survivability, and capacity to cause disease, highlighting the dynamic nature of the host-pathogen interaction and the potential for *C. neoformans* to adapt to different host microenvironments.

## Discussion

Cryptococcosis, primarily caused by *C. neoformans* and *C. gattii*, represents a significant opportunistic fungal infection often associated with immunocompromised states, such as HIV infection, as well as other underlying disorders including immunosuppressant use, transplantation, cancer, and diabetes mellitus. While studies on cryptococcosis in China have largely focused on HIV-uninfected patient [17], the current study expands on this by systematically evaluating the impact of genetic and environmental factors on the virulence-related phenotypes of *C. neoformans* isolates from a broader range of sources. Specifically, we compared strains isolated from HIV-infected and HIV-uninfected patients, as well as from natural environmental reservoirs, to explore how host environments influence the genetic diversity and pathogenicity of *C. neoformans*.

As previously reported, host-specific factors such as IgM levels and androgen exposure may modulate the virulence of *C. neoformans* by influencing fungal stress-response pathways [58–60]. However, a primary limitation of our study is the incompleteness of clinical metadata, particularly the absence of systematic immunological profiling – including IgM titers and hormonal levels. This constraint impairs our ability to establish robust statistical correlations between these host variables and fungal virulence characteristics.

In addition to these acknowledged limitations, Table 1 identifies three clinical parameters that exhibit significant differences between HIV-i and HIV-u groups: CD4+ T-cell counts, body temperature and fever patterns, and neurological complications (e.g. seizures, brain herniation, cerebellar dysfunction). Below, we explore their potential implications for *C. neoformans* pathogenesis. In HIV-infected patients, severe CD4+ T-cell depletion compromises cell-mediated immunity, thereby enabling *C. neoformans* to enhance virulence through mechanisms such as capsule thickening, melanin production, and micro-cell morphotype formation, all of which facilitate immune evasion and systemic dissemination. Additionally, increased capsule shedding further suppresses T-cell responses, reinforcing immune subversion [61,62]. Similarly, body temperature plays a crucial role in fungal adaptation, as *C. neoformans* exhibits strain-specific thermotolerance, dynamically regulating temperature-responsive genes and capsule components to optimize host colonization. Notably, capsule thickness has been shown to correlate with fever in HIV patients [62], while fungal persistence varies by tissue temperature, favoring cooler niches like the testes over warmer CNS compartments [63–65]. However, minor physiological temperature fluctuations (~37.1–37.6°C) appear insufficient to drive significant virulence evolution. Furthermore, neurological complications such as seizures, brain herniation, and cerebellar dysfunction, though commonly associated with cryptococcal meningitis, have not been directly linked to fungal virulence. Instead, elevated intracranial pressure, driven by micro-cell proliferation and capsule shedding, likely contributes to neural tissue compression and associated symptoms [62]. Nevertheless, no studies have conclusively demonstrated a direct relationship between these complications and specific *C. neoformans* pathogenic traits.

In our study, we observed significant correlations and trends across the three different environments, suggesting that host environments play a crucial role in shaping the virulence of *C. neoformans* isolates. Strikingly, strains isolated from HIV-infected patients exhibited markedly reduced virulence compared to those from HIV-uninfected individuals and environmental sources, highlighting the plasticity of *C. neoformans* pathogenicity in response to differing host conditions. This observation points to the ability of *C. neoformans* to adapt its virulence mechanisms based on the host’s immune status, suggesting a dynamic interaction where the fungus modifies its pathogenic potential to enhance survival and persistence in varied environments. Whole-genome sequencing and GWAS analysis further support this view, revealing that the high frequency of hotspot mutations in carbohydrate anabolism genes may underlie the observed differences in virulence. These mutations likely play a key role in driving the differential pathogenicity of isolates from HIV-uninfected versus HIV-infected patients.

Broad epidemiology and molecular typing studies of *C. neoformans* and *C. gattii* species complexes have been reported, revealing that genotypic distributions are closely tied to geographic regions [13]. Initially, we aimed to investigate the genotype–phenotype correlation by analyzing strains with different sequence types (STs). However, contrary to expectations, our results revealed that all 105 isolates belonged to the molecular-type AFLP1/VNI, with ST5 being the most prevalent sequence type (*n* = 94; 89.5%). While other STs were also identified using MLST, the dominance of ST5 precluded a comparative analysis of virulence factor production between isolates from different STs. To our surprise, this finding aligns with observations from other Asian countries, where ST5 is also the predominant sequence type responsible for human cryptococcosis [66]. A distinct genotypic characteristic of Chinese *C. neoformans* isolates was that the prevalence of ST5, which was the most common genotype found in clinical isolates from both HIV-infected and -uninfected samples. In contrast, the genotypes of natural isolates, such as those from pigeon droppings, exhibited significantly greater diversity. Moreover, available data suggest that the genotypic diversity of isolates from HIV-infected patients is more limited compared to those from HIV-uninfected individuals. However, further validation through larger isolate collections and comprehensive data analysis is necessary to fully support this observation.

Although strains with different genotypes shared similar mating types, we observed significant differences in melanin production and capsule formation. Strains from HIV-uninfected patients produced notably more melanin than those from HIV-infected patients and natural sources. This suggests that cryptococcal strains increase melanin biosynthesis in response to a stronger immune system, whereas in HIV-infected patients, where immune function is compromised, this response is diminished. This pattern indicates that the pathogen adapts its virulence mechanisms based on the host’s immune strength, with heightened melanin production in HIV-uninfected individuals likely aiding immune evasion, while reduced virulence in HIV-infected patients reflects adaptation to an immunosuppressed environment. Considering the prevalence of L-DOPA in the central nervous system [15], our results suggest that increased melanin production in strains isolated from HIV-uninfected patients could occur *in vivo*, thereby promoting virulence and potentially leading to higher mortality. Additionally, capsule formation in different strains showed similar patterns. A previous study indicated that capsule size in cryptococcal cells varies significantly between species and individuals, further supporting the idea that capsule size may influence virulence and pathogenic outcomes [62]. As expected, we observed the smallest size of capsule size in strains from HIV-infected patients, likely reflecting their ability to more easily cross biological barriers and disseminate within the brain of immunocompromised patients. However, the relationships between capsule size and virulence remains controversial. One study reported that *C. neoformans* isolates with higher capsules were associated with slower fungal clearance and increased intracranial pressure, suggesting that capsule size may not always correlate directly with pathogenicity or immune evasion [61]. However, this notion has been contested by other studies, which found that *C. neoformans* isolates with smaller capsules were more virulent and resulted in a higher fungal load in the brain [41]. To verify that *in vitro* capsule and melanin production correlate with pathogenicity, we examined additional virulence-related traits, such as *C. neoformans* survival in human cerebrospinal fluid (CSF), macrophage uptake, and morbidity in a mouse model of cryptococcosis. The in vitro, ex vivo, and in vivo data strongly support the role of the host environment in shaping the virulence of *C. neoformans*. It is worth mentioning that our findings align closely with the study by Robertson *et al*., which also emphasized the significant influence of the host’s immune state on the pathogen’s virulence evolution [61]. Given the impaired immune system in HIV-infected patients, it is reasonable to conclude that virulence may not play a significant role in the establishment of a successful infection. In contrast, for colonization in relatively healthy individuals, such as HIV-negative patients, cryptococcal strains must evolve to enhance their virulence traits in order to effectively combat the host’s immune defenses.

Mutation is a fundamental source of genetic variation necessary for adaptation, and typically, the rate of genomic mutations is optimized to enhance the organism’s ability to adapt. We initially hypothesized that frequent genetic variations might drive the observed differences in virulence among strains from distinct groups. However, our findings did not support this hypothesis. In fact, the attenuation of virulence and the phenotypic changes observed in strains from HIV-infected patients could not be largely explained by high-frequency genomic alterations. This aligns with a previous whole-genome sequencing study on 32 isolates from 18 South African patients with recurrent cryptococcal meningitis, which found that only a limited number of genetic changes – such as single nucleotide polymorphisms (SNPs), small insertions/deletions (indels), and variations in copy number, were identified between incident and relapse isolates [67]. However, we did observe genetic variations in a selected group of genes between the two groups. Specifically, the isolates from HIV-uninfected group exhibited frequent mutations in genes associated with carbohydrate metabolism. Recent studies have shown that these genes play a crucial role in supporting *C. neoformans* growth and regulating the production of key virulence factors, such as the polysaccharide capsule, melanin, and secreted enzymes [68,69]. This suggests an increase in virulent phenotypes in the HIV-uninfected group compared to those from the HIV-infected group. Secondly, we found that the HIV-u group carried mutations in key genes involved in pathways such as Vesicle-mediated transport. We speculate that they may be related to capsule and melanin formation in the strains, due to they were not directly linked to phenotypic traits, but some genes may be associated with mRNA processing in the HIV-i group. However, further experimental validation is still needed. Together, these genetic mutations likely reflect an adaptive response to the host environment, driving the evolution of fungal virulence.

In conclusion, through the comprehensive integration of genotyping, pathogenic phenotyping, and comparative genomics, our study represents the first systematic effort to characterize the phenotypic and virulence-related properties of *C. neoformans* strains derived from three distinct host environments: HIV-uninfected individuals, HIV-infected patients, and natural sources. Our findings highlight the critical role of host environmental factors in shaping fungal virulence, demonstrating that the attenuation of pathogenicity observed in strains from HIV-infected patients is likely driven by the unique conditions of the immunocompromised host. In contrast, strains from HIV-uninfected individuals exhibited heightened virulence, suggesting that the pathogen’s ability to adapt to varying host immune landscapes plays a key role in its pathogenic success. Moreover, we provide evidence that genetic variations, particularly in genes associated with carbohydrate metabolism, are central to the adaptive evolution of *C. neoformans* virulence, facilitating immune evasion and supporting colonization in diverse host environments. These insights not only challenge our understanding of *C. neoformans* pathogenesis but also highlight the dynamic interaction between genetic and environmental factors in driving fungal evolution. Moving forward, more detailed biochemical, molecular, and immunological studies are essential to fully understand the mechanisms by which these factors regulate pathogenicity and contribute to invasive cryptococcosis. Such investigations will be critical in advancing therapeutic strategies targeting these adaptive mechanisms and may provide novel avenues for combating this persistent and opportunistic fungus.

## Supplementary Material

Supplemental_material clean file.docx

## Data Availability

The authors declare that all the data supporting the findings of this study are available within the article and its supplementary information files. Raw data and supplemental material for this study are available at https://doi.org/10.6084/m9.figshare.27991232. Whole-genome sequencing data have been deposited in NCBI under BioProject ID: PRJNA1182044.

## Preprint

A preprint version of this article is available at https://doi.org/10.1101/2021.10.15.464490.
