# Supplementary material for: Intrinsic and extrinsic factors affecting the evolution of virulence in the HIV-associated opportunistic human fungal pathogen Cryptococcus neoformans
Source: Virulence. 2025 Aug 14;16(1):2546067. doi: 10.1080/21505594.2025.2546067 (PMC12363409; doi:10.1080/21505594.2025.2546067)
Supplement: Supplemental_material clean file.docx [file KVIR_A_2546067_SM9477.docx]

**Supplemental material**

**Figure S1.** The mating types of all 105 cryptococcal isolates were determined by multiplex PCR. Genomic DNA was extracted from each isolate, and the highly conserved mating type locus alleles were identified through PCR amplification with specific primers listed in **Table S6**. Two control strains, H99 (MATα) and JEC20 (MATa), were included for comparison to ensure accurate identification of mating types.

**Figure S2.** Melanin biosynthesis of all 105 isolates. An individual strain from each of the HIV-i group **(A)**, HIV-u group **(B)** or Env group **(C)** isolates was incubated at 30℃ in minimal media supplemented with 1mM L-DOPA. Images were captured after 48 hours of incubation. The melanized *C. neoformans* strain H99 and the non-melanized strains, including *Candida albicans* SC5314 and *C. neoformans* JEC20, served as positive and negative controls, respectively.

**Figure S3.** Capsule formation in *C. neoformans* isolates from the HIV-infected patients. Each isolate was incubated at 30℃ for 48 hours under two different conditions: un-induced (YPD broth) and induced (10% Sabouraud medium buffered to pH 7.3 with 50 mM MOPS). Following incubation, cells were stained with India ink, and the capsule thickness of each colony was measured. Scale bars represent 10 μM.

**Figure S4.** Capsule formation in *C. neoformans* isolates from HIV-uninfected individuals. The procedures were carried out as described in **Figure S3**. Scale bars represent 10 μM.

**Figure S5.** Capsule formation in *C. neoformans* strains isolated from natural resources (pigeon excreta). The procedures were conducted as described in **Figure S3**, using strains isolated from pigeon excreta. Scale bars represent 10 μm.

**Figure S6.** Survival curves of *G. mellonella* infected with *C. neoformans* isolates from each of the three groups. The *G. mellonella* (n=20) were infected with 5×10^5^ CFUs of individual strains from the HIV-infected, HIV-uninfected, and natural environment groups was monitored. Larvae were observed for signs of severe morbidity, and survival was tracked over time.

**Figure S7.** Kaplan-Meier survival curves of mice infected with individual strains. Groups of female C57BL/6 mice (n=8 per group) were inoculated intranasally with 1×10^5^ CFUs of the indicated strain and monitored for progression to severe morbidity. Three representative isolates from each of the three groups were used. For comparison, the H99 strain was included as a control. Note: All *C. neoformans* strains, including the control strain H99 and all 9 tested isolates, were tested for pathogenicity in a single experiment. ****p* < 0.001, by Log-rank test.

**Figure S8.** Comparative analysis of *C. neoformans* isolates from different groups (ENV, HIV-i, and HIV-u). **(A)** Phylogenetic tree constructed based on SNPs from whole-genome sequences by using IQ-TREE. The resulting phylogeny was visualized using ggtree in R (v4.4.0). Phylogenetic tree was categorized into three groups: 50 from the HIV-i group (blue), 38 from the HIV-u group (red), and 4 from the ENV group (green). Metadata including group, melanin production (dark blue), capsule size (dark red), and survival (dark green) values are shown as concentric heatmap tracks. All branches are rendered at equal length for clarity, with actual evolutionary distances higher than 1 labeled on branch. Each circle represents an individual isolate, with the corresponding sequence type (ST) indicated by differently colored circles along the tree’s outer edge. **(B)** Principal Component Analysis (PCA) plot based on genetic variation among the isolates. Each point represents a single isolate, color-coded according to group. The clustering patterns indicate distinct genetic differentiation between the HIV-i and HIV-u group.

**Figure S9.** Summary of genomic variants identified from whole-genome sequencing of selected *C. neoformans* isolates. **(A)** A total of 92 isolates, including 50 from the HIV-i group, 38 from the HIV-u group, and 4 from the ENV group, were sequenced. The number of variants identified in each gene locus is presented, with genes containing at least 10 variants in the HIV-i and HIV-u groups, and at least 5 variants in the ENV group, shown in the magnified inset panel. **(B)** All sequenced isolates from both groups showed no correlation between the number of variants and gene length per base pair in each gene. **(C)** Distribution of variants across the genomes of sequenced isolates. Notably, a cluster of genes that were identical in both groups had significantly high numbers of variants.

**Figure S10. Circos plot depicting the genome-wide genetic diversity of three groups of *C. neoformans*.** The outermost track shows chromosome names. From outside to inside, each ring represents one mutation type: nonsynonymous SNPs, synonymous SNPs, deletions, and insertions. Histograms in each track show the total number of each mutation type in 50 kb sliding windows. Lines in different colors represent different groups: HIV-i group (blue), HIV-u group (red), and ENV group (green). Each line plot shows mutation density ranging from 0 to 2000 per window. SNPs were identified by reference-based mapping and variant calling using the GATK pipeline.

**Figure S11. Quantile-quantile (Q-Q) plots of p-values for genome-wide association studies (GWAS) examining genes associated with capsule formation, melanin production, and survival.** The x-axis represents the expected –log_10_ (p-values), while the y-axis shows the observed –log_10_ (p-values). The red dashed line indicates the null hypothesis observed and expected values align. Deviations from the line suggest potential associations with the respective traits.

**Table S1.** The Source and Sequence Types (ST) of all 105 *Cryptococcus* isolates

**Table S2**. Primers used in this study

**Table S3.** Quality control of the whole-genome sequencing data derived from the selected 105 *C. neoformans* isolates

**Table S4.** Summaries of the sequencing reads for each of the 105 *C. neoformans* isolates. Note: the reads were mapped to the reference genome of *Cryptococcus neoformans var. grubii* H99

**Table S5.** Summaries of genetic variants identified in each genome of the 105 sequenced *C. neoformans* isolates

**Table S6.** Characterization of major genes harboring specific variations in HIV-u and HIV-i groups, respectively
